# Photorefractive Keratectomy for Residual Myopia after Myopic Laser In Situ Keratomileusis

**DOI:** 10.1155/2017/8725172

**Published:** 2017-01-12

**Authors:** Kamal A. M. Solaiman, Sameh M. Fouda, Ashraf Bor'i, Haitham Y. Al-Nashar

**Affiliations:** Ophthalmology Department, Zagazig University, Zagazig, Egypt

## Abstract

*Purpose*. To evaluate the safety, efficacy, and predictability of photorefractive keratectomy (PRK) on the corneal flap for correction of residual myopia following myopic laser in situ keratomileusis (LASIK).* Patients and Methods*. A retrospective study on eyes retreated by PRK on the corneal flap for residual myopia after LASIK. All eyes had no enough stroma after LASIK sufficient for LASIK enhancement. Data included spherical equivalent (SE), uncorrected and best corrected visual acuity (UCVA and BCVA), central pachymetry, corneal higher order aberrations (HOAs), corneal hysteresis (CH), corneal resistance factor (CRF), and corneal haze.* Results*. The study included 64 eyes. Before PRK, the mean central pachymetry was 400.21 ± 7.8 *μ*m, the mean SE was −1.74 ± 0.51 D, and the mean UCVA and BCVA were 0.35 ± 0.18 and 0.91 ± 0.07, respectively. 12 months postoperatively, the mean central corneal thickness was 382.41 ± 2.61 *μ*m, the mean SE was −0.18 ± 0.32 D (*P* < 0.01), and the mean UCVA and BCVA were 0.78 ± 0.14 (*P* = 0.01) and 0.92 ± 0.13 (*P* > 0.5), respectively. The safety index was 1.01 and the efficacy index was 0.86. No significant change was observed in corneal HOAs.* Conclusions*. Residual myopia less than 3 D after LASIK could be safely and effectively treated by PRK and mitomycin C with a high predictability. This prevents postoperative ectasia and avoids the flap related complications but has no significant effect on HOAs.

## 1. Introduction

An important goal in refractive surgery is to abolish postoperative refractive error and to decrease the complications of retreatment if needed [1]. The evolution of excimer laser refractive surgery to eliminate ametropia has resulted in increasingly accurate and predictable results. However, retreatment for residual ametropia may be required to fine tune the outcome [2–4].

Retreatment, which is typically performed on 10 to 20% of patients who undergo laser in situ keratomileusis (LASIK), is considered by the patients to reflect failure of the original procedure; therefore retreatment should be precise to address patient satisfaction and maintain safety [5]. However, LASIK may not be safe as a retreatment, as an increased number of eyes with ectasia were reported following LASIK enhancement [6, 7]. Different retreatment options are available for corneas with compromised thickness, such as treatment of the flap undersurface, laser-assisted subepithelial keratectomy (LASEK), and trans- or subepithelial photorefractive keratectomy (PRK) [8, 9].

Many studies [10, 11] have reported that performing excimer laser surface ablation (PRK) reduces the risk of ectasia by preserving the corneal stroma as much as possible and avoids the flap-related complications caused by either original flap manipulation or new flap creation. Furthermore, corneal wavefront-guided PRK can reduce flap-induced higher order aberrations, resulting in better outcomes [10, 11]. The aim of this study was to evaluate the safety, predictability, and efficacy of PRK on the corneal flap for correction of residual myopia following LASIK.

## 2. Patients and Methods

This is a retrospective study that included eyes with a residual myopia and/or astigmatism after LASIK procedure. The inclusion criteria included residual mean spherical equivalent between −0.75 D  and  −2.75 D, stable refraction for at least 6 months before PRK, and calculated postoperative corneal thickness < 380 *μ*m (which is not safe for LASIK retreatment). Exclusion criteria included eyes with post-LASIK corneal ectasia, flap striae, central islands, and lenticular myopia and those unavailable for 12 months followup. The study followed the tenets of Helsinki Declaration and an informed written consent was obtained from each patient before PRK. All PRK procedures were performed between February 2013 and April 2015 in a private eye center in Egypt.

All patients received preoperative full ophthalmic examination using slit lamp examination, manifest and cycloplegic refractions, uncorrected visual acuity (UCVA), best corrected visual acuity (BCVA), applanation tonometry, and dilated funduscopy. Visual acuity was determined using a standard acuity chart at 6 meters. Dual scan corneal tomography which combines rotating Scheimpflug imaging with Placido disc corneal topography using a Sirius imager (Schwind Eye-Tech Solutions, Germany) was performed to determine the residual corneal thickness and HOAs.

After topical surface anesthesia, the corneal epithelial layer was removed by laser using Trans-PRK mode. The calculated epithelial thickness to be removed ranged between 55 and 65 microns (thinner in the center) and the epithelial ablation profile was adjusted by complex software to the degree of error to be corrected, the corneal wavefront map, and the default optical zone. Then, a corneal wavefront-guided surface ablation of the flap was performed using a Schwind Amaris Excimer Laser (Schwind Eye-Tech Solutions, Germany) with targeted refraction of emmetropia. The optic zone diameter was 6 mm with a 1 mm transition zone. Following photoablation, 0.02% (0.2 mg/cc) mitomycin C was applied for 1 minute. The cornea was then irrigated copiously with 25 cc of balanced salt solution to wash out the residual mitomycin C. A bandage contact lens was placed at the end of the procedure. All operated eyes received postoperative treatment with 0.3% gatifloxacin 4 times a day for one week together with 1% prednisolone acetate twice a day to be increased to 4 times/day for 1-2 weeks after healing of the epithelium and replaced by fluorometholone 0.25% 4 times/day for 3–6 months based on the degree of corneal haze and intraocular pressure. All patients were advised to constantly wear protective sunglasses outdoors during daytime.

Biomechanical properties of the cornea were determined in all eyes using Ocular Response Analyser (ORA, Reichert, Depew, New York). Corneal hysteresis (CH) and corneal resistance factor (CRF) were measured in all patients preoperatively and at every follow-up visit.

Patients' data were reported at 1, 3, 6, and 12 months postoperatively. The main outcome measures included refractive predictability and stability, residual refractive error, UCVA, BCVA, and HOAs as well as any reported complication as corneal haze and corneal ectasia. Corneal haze was graded on a scale of 0 to 4 according to Fantes classification [12]. The efficacy and safety indices were calculated as follows:* efficacy index* = mean postoperative UCVA/mean preoperative BCVA, and* safety index* = mean postoperative BCVA/mean preoperative BCVA.

Statistical analysis was performed via paired *t* test using SPSS for Windows (version 16, SPSS, Chicago). For all analyses, a *P* value of < 0.05 was considered significant statistically.

## 3. Results

Sixty-four eyes of 52 patients (26 in males and 38 in females) with a mean age 33.7 ± 6.4 years (range 23–46 years) were eligible to be included in this retrospective study. Eyes had a mean myopic spherical equivalent 9.21 ± 2.43 D (range 6.75–12.25 D) before LASIK procedure. Residual myopia after LASIK was due to undercorrection and/or myopic regression. The mean duration between LASIK and PRK was 17.34 ± 5.41 months (range 6–27 months). Just before PRK procedure, the mean UCVA was 0.35 ± 0.18 (range 0.1–0.7), the mean BCVA was 0.91 ± 0.07 (range 0.8–1.00), and the mean central corneal thickness was 400.21 ± 7.8 *μ*m (range 389–412 *μ*m). The residual mean myopic spherical equivalent was 1.74 ± 0.51 D (range 0.75–2.75 D). The mean astigmatism was 0.76 ± 0.44 D (0.00–3.00 D).

Postoperative data were reported at 1, 3, 6, and 12 months. The residual mean spherical equivalent error showed statistical significant improvement (*P* < 0.05) to −0.23 ± 0.45 D (range +0.75 to −1.25 D) at 1 month, −0.19 ± 0.4 D (range +0.75 to −1.00 D) at 3 months, −0.20 ± 0.35 D (range +0.50 to −0.75 D) at 6 months, and −0.18 ± 0.32 D (range +0.50 to −0.50 D) at 12 months. By the end of the follow-up duration, the mean astigmatism was 0.61 ± 0.23 D (0.25–2.25 D) (*P* > 0.05), the mean surgically induced astigmatism was 0.31 ± 0.11 D (0.25–0.75 D), the mean magnitude of error was 0.72 ± 0.11 D (0.58 ± 0.88), the mean angle of error was −9.5 ± 3.4 degrees, and the correction factor for astigmatism was 0.56 ± 0.22.

The mean UCVA was significantly improved at 1, 3, 6, and 12 months postoperatively to 0.71 ± 0.14 (range 0.4–1.00) (*P* = 0.03), 0.76 ± 0.12 (range 0.6–1.00) (*P* = 0.01), 0.77 ± 0.13 (range 0.6–1.00) (*P* = 0.01), and 0.78 ± 0.14 (range 0.6–1.00) (*P* = 0.01), respectively. The postoperative changes in UCVA were not significant statistically (*P* > 0.05) between all follow-up visits. UCVA was nearly stabilized after 6 months up to 12 months. The mean BCVA at 1, 3, 6, and 12 months postoperatively were 0.90 ± 0.07 (range 0.7–1.00), 0.90 ± 0.17 (range 0.7–1.00), 0.91 ± 0.13 (range 0.8–1.00), and 0.92 ± 0.13 (range 0.8–1.00), respectively. The differences between pre- and postoperative values were not significant statistically (*P* > 0.05) at all follow-up visits. BCVA was unchanged or improved in all cases, and no eye had lost line of BCVA after PRK. The efficacy index was 0.86 and the safety index was 1.01 at the 12-month follow-up visit.

HOAs were measured for 5 mm pupil. Preoperatively, the mean total corneal HOAs were 0.26 ± 0.14 *μ*m (range 0.23–0.47 *μ*m), the mean coma aberrations 0.28 ± 0.17 *μ*m (range 0.24–0.31 *μ*m), and the mean spherical aberrations 0.26 ± 0.11 *μ*m (range 0.27–0.24 *μ*m). Postoperatively, the mean total corneal HOAs were 0.28 ± 0.11 *μ*m (range 0.21–0.50 *μ*m), the mean coma aberrations 0.26 ± 0.50 *μ*m (range 0.20–0.32 *μ*m), and the mean spherical aberrations 0.23 ± 0.13 *μ*m (range 0.22–0.24 *μ*m), (*P* > 0.05).

The mean ablation depth was 26.27 ± 5.73 *μ*m, and the mean central corneal thickness 12 months after PRK was 382.41 ± 2.61 *μ*m (range 379–384 *μ*m). CH before PRK ranged from 8.5 to 9.1 with a mean of 8.81 ± 0.17. No significant change in CH was observed during the follow-up period. CH at 1, 3, 6, and 12 months postoperatively was 8.78 ± 0.18 (range 8.5–9.1) (*P* > 0.5), 8.75 ± 0.18 (range 8.4–9.0) (*P* > 0.5), 8.7 ± 0.17 (range 8.3–9.0) (*P* > 0.5), and 8.7 ± 0.2 (range 8.3–9) (*P* > 0.5), respectively. The preoperative CRF was 7.34 ± 0.3 (range 7-8). No significant change in CRF was observed over the follow-up period. CRF at 1, 3, 6, and 12 months postoperatively was 7.27 ± 0.27 (range 7–7.9) (*P* > 0.5), 7.33 ± 0.29 (range 7–7.9) (*P* > 0.5), 7.32 ± 0.3 (range 77.9) (*P* > 0.5), and 7.3 ± 0.25 (range 7–7.8) (*P* > 0.5), respectively.  Table 1 shows the preoperative and the 12-month postoperative data.

Complete reepithelialization of the cornea and contact lens removal was achieved within the first few days (2 to 5 days) postoperatively. Grades 1 to 2 corneal haze was observed in 25 eyes (39%) and this haze disappeared during the first month postoperatively in 23 eyes. In one eye the corneal haze disappeared by the 3rd month and in one eye it persisted up to the 6th month postoperatively. Corneal ectasia was not detected in any of the eyes of the study. No vision-threatening complications were detected in all eyes by the end of this study.

## 4. Discussion

Since the introduction of photorefractive keratectomy (PRK) in the year 1987 and laser in situ keratomileusis (LASIK) in the year 1990, LASIK has been preferred over PRK due to painless and rapid visual rehabilitation as well as too much less postoperative corneal haze than PRK [13, 14]. However, PRK is still having its own indications when LASIK is not safe to be performed. One of these indications is the absence of enough stromal thickness under the flap sufficient for full correction of residual ametropia by LASIK enhancement.

Residual ametropia (undercorrection or overcorrection) is the most common complication after LASIK [5]. It has been reported that regression of myopia is a universal phenomenon after excimer laser correction of myopia and is greater for higher corrections [15]. The reasons for myopic regression could be epithelial hyperplasia, corneal steepening because of thinning, change in corneal biomechanics, and lenticular sclerosis [15]. Retreatment of post-LASIK ametropia should be considered if it troubles the patient [16, 17]. The first choice for retreatment is LASIK enhancement by flap lifting and laser application to the underlying stroma. But this is not safe in eyes with insufficient residual corneal stromal thickness [18, 19]. In such cases, excimer laser superficial keratectomy techniques should be considered, for example, photorefractive keratectomy (PRK), epithelial laser in situ keratomileusis (Epi-LASIK), or laser subepithelial keratectomy (LASEK) [14, 20, 21]. Introduction of wavefront technology has raised the chances for correction of residual errors and other complications after LASIK [22]. Lee et al. [23] reported that PRK is highly effective and safe for patients with previous LASIK and in whom the surgeon would prefer not to do a flap-lift enhancement.

This study included 64 eyes having residual myopia and thin corneas after myopic LASIK correction. All eyes were corrected by wavefront-guided PRK combined with MMC application over the flap surface. Trans-PRK mode was used to avoid the risk of flap displacement that could happen with mechanical removal of epithelium. Another advantage is the postoperative faster healing of epithelium and less possibility of corneal haze if alcohol was used for removal of epithelium. On the other hand, any change in corneal epithelium in those previously treated eyes with LASIK could affect the results, especially HOAs and astigmatism.

At 12 months after PRK, the mean SE showed statistically significant improvement. However, the improvement in astigmatism was less than the improvement achieved in spherical myopia. By the end of the follow-up period, 62 eyes (96.9%) were within ±0.5 D. This indicates good predictability of PRK for residual myopia following LASIK. It has been reported that predictability is better in low myopia than in moderate or high myopia [24]. All eyes in this study had low myopic error. PRK studies with prolonged followup have reported that refractive stability was achieved by 6 months to 1 year and was maintained up to 12–14 years without significant late regression, hyperopic shift, or fluctuation [24–27]. In this study, no myopic shift was reported after 6 months and up to 12 months.

The mean UCVA showed significant improvement from 0.35 ± 0.18 preoperatively to 0.78 ± 0.14 at the end of the follow-up period. Previous studies reported that eyes with lower myopia tend to achieve higher postoperative UCVA [28–30]. This was not the case in this study although the mean spherical myopic error was low. This is most probably because the original mean error before LASIK was high (−9.21 ± 2.43 D). By the end of the study, the mean BCVA did not show a change and no eye had lost any line. However, both UCVA and BCVA were less at 1 month due to corneal haze but they improved gradually after that and become almost stable after 6 months. One study [31] suggested a prolonged healing time after PRK in a lamellar flap compared with the healing time for an uncut cornea. In the small errors as in this study, large part of the laser treatment occurred in the acellular Bowman's membrane. This could explain the prolonged healing time and mild corneal haze reported in this study. On the other hand, Güell et al. [10] corrected regression after LASIK using intraepithelial PRK in which Bowman's membrane is left intact and there is no stromal healing, but only 52.4% of their cases were within ±0.50 D of emmetropia after 6 months compared to 90.6% in this study. The efficacy index in this study was 0.86 at 1 year which is better than that reported by Koshimizu et al. [32] (0.74) and by Alio et al. [15] (0.81) and this is most probably because they reported their efficacy index at a longer follow-up period (10 years) after PRK. The safety index in this study was 1.01 at 12 months, indicating safety of PRK for residual myopia following LASIK. The results of this study are matching with the results of Beerthuizen and Siebelt [31] who treated 18 eyes having residual ametropia after LASIK via wavefront-guided PRK on the flap. They reported a BCVA of 0.95 ± 1.0 and a safety index of 1.0 after 12-month followup. Shaikh et al. [33] also have evaluated the safety and efficacy of PRK on corneas previously treated with LASIK in 15 eyes. They concluded that PRK is a safe procedure that could reduce refractive error and improve both UCVA and BCVA in corneas previously treated with LASIK surgery. The changes in corneal HOAs after PRK were not statistically significant in spite of using corneal wave front guided PRK. This could be explained by the presence of another interface below the flap.

Corneal haze is more common after PRK than after LASIK due to more activation of corneal fibroblasts and keratocytes following PRK [34]. To reduce postoperative corneal haze in this study, PRK was delayed for at least 6 months after LASIK to allow keratocyte activity to recede. Also, no alcohol was used for removal of epithelium and intraoperative MMC 0.02% was applied for 1 minute to corneal stroma immediately after laser ablation. The original protocol of intraoperative MMC suggested 2 minutes exposure time for modulation of corneal wound healing and prevention of corneal haze after PRK [35]. However, the MMC exposure time has been reduced down to 15 seconds depending on the degree of error to be corrected [36]. In this study, the exposure time of MMC was 1 minute. In spite of this, grades 1-2 corneal haze was observed in 25 eyes (39%). Corneal haze disappeared during the first postoperative month in 23 eyes and disappeared by the 3rd month in 1 eye and in one eye it persisted up to the 6th month postoperatively. Beerthuizen and Siebelt [31] reported prolonged grade 1 corneal haze that persisted up to 6 months in 1 eye in their study that included 18 eyes treated with PRK for residual ametropia after LASIK. In another prospective study [37] on 16 eyes treated with PRK and mitomycin C (50 seconds) for residual error following LASIK, none of the eyes developed corneal haze of any degree during 6-month followup. A study reported that exposure to high ultraviolet radiation level may increase the risk of late-onset corneal haze in eyes with moderate to high myopia [38]. However, no late-onset corneal haze was reported in this study which was performed in a subtropical area with high ultraviolet radiation. This could be attributed to the postoperative constant use of sunglasses outdoors during daytime by all patients.

CH and CRF are biomechanical properties of the cornea that reflect its viscoelastic properties [39]. In this study, no statistically significant differences were found between the values of CH and CRF before and after PRK and throughout the follow-up period. This stability could be due to application of the excimer laser over the flap without ablating the residual stromal bed. No postoperative corneal ectasia and no vision-threatening complications were reported in this study.

In conclusion, residual myopia less than 3 D after LASIK could be safely and effectively treated by PRK and mitomycin C with high predictability. This prevents postoperative ectasia and avoids the flap-related complications caused by manipulation of the original flap or creation of a new flap. On the other hand, it has no significant effect on corneal HOAs.

## Figures and Tables

**Table 1:** Preoperative and 12-month postoperative data of eyes included in the study.

	Before PRK Mean ± SD (range)	12 months after PRK Mean ± SD (range)	*P* value
Spherical error	−1.74 ± 0.51 D (−0.75–0.75 D)	−0.18 ± 0.32 D (+0.50 to −0.50 D)	<0.05^*∗*^
Astigmatism	−0.76 ± 0.44 D (0.00–3.00 D)	−0.61 ± 0.23 D (0.25–2.25 D)	>0.05
Uncorrected visual acuity	0.35 ± 0.18 (0.1–0.7)	0.78 ± 0.14 (0.6–1.00)	<0.05^*∗*^
Best corrected visual acuity	0.91 ± 0.07 (0.8–1.00)	0.92 ± 0.13 (0.8–1.00)	>0.05
Central corneal thickness	400.21 ± 7.8 *µ*m (389–412 *µ*m)	382.41 ± 2.61 *µ*m (379–384 *µ*m)	>0.05
Corneal hysteresis	8.81 ± 0.17 (8.5–9.1)	8.70 ± 0.20 (8.3–9.0)	>0.05
Corneal resistance factor	7.34 ± 0.3 (7.0–8.0)	7.3 ± 0.25 (7.0–7.8)	>0.05
Total high-order aberrations	0.26 ± 0.14 *µ*m (0.23–0.47 *µ*m)	0.28 ± 0.11 *µ*m (0.21–0.50 *µ*m)	>0.05
Coma	0.28 ± 0.17 *µ*m (0.24–0.31 *µ*m)	0.26 ± 0.15 *µ*m (0.20–0.32 *µ*m)	>0.05
Spherical aberrations	0.26 ± 0.11 *µ*m (0.27–0.24 *µ*m)	0.23 ± 0.13 *µ*m (0.22–0.24 *µ*m)	>0.05

*∗* means significant result.
